# Prevalence of Anal Human Papillomavirus Infection and Risk Factors among HIV-positive Patients in Tokyo, Japan

**DOI:** 10.1371/journal.pone.0137434

**Published:** 2015-09-14

**Authors:** Naoyoshi Nagata, Kazuhiro Watanabe, Takeshi Nishijima, Kenichi Tadokoro, Koji Watanabe, Takuro Shimbo, Ryota Niikura, Katsunori Sekine, Junichi Akiyama, Katsuji Teruya, Hiroyuki Gatanaga, Yoshimi Kikuchi, Naomi Uemura, Shinichi Oka

**Affiliations:** 1 Department of Gastroenterology and Hepatology, National Center for Global Health and Medicine, Tokyo, Japan; 2 AIDS Clinical Center, National Center for Global Health and Medicine, Tokyo, Japan; 3 BML, Tokyo, Japan; 4 Ohta Nishinouchi Hospital, Fukushima, Japan; 5 Department of Gastroenterology and Hepatology, National Center for Global Health and Medicine, Kohnodai Hospital, Chiba, Japan; State University of Maringá/Universidade Estadual de Maringá, BRAZIL

## Abstract

**Background:**

Oncogenic human papillomavirus (HPV) infection, particularly multiple HPV types, is recognized as a necessary cause of anal cancer. However, a limited number of studies have reported the prevalence of anal HPV infection in Asia. We determined the prevalence, genotypes, and risk factors for anal HPV infection in Japanese HIV-positive men who have sex with men (MSM), heterosexual men, and women.

**Methods:**

This cross-sectional study included 421 HIV-positive patients. At enrollment, we collected data on smoking, alcohol, co-morbidities, drugs, CD4 cell counts, HIV RNA levels, highly active anti-retroviral therapy (HAART) duration, sexually transmitted infections (STIs), and serological screening (syphilis, hepatitis B virus, *Chlamydia trachomatis*, *Entamoeba histolytica*). Anal swabs were collected for oncogenic HPV genotyping.

**Results:**

Oncogenic HPV rate was 75.9% in MSM, 20.6% in heterosexual men, and 19.2% in women. HPV 16/18 types were detected in 34.9% of MSM, 17.7% of heterosexual men, and 11.5% of women. Multiple oncogenic HPV (≥2 oncogenic types) rate was 54.6% in MSM, 8.8% in heterosexual men, and 0% in women. In univariate analysis, younger age, male sex, MSM, CD4 <100, HIV viral load >50,000, no administration of HAART, and having ≥2 sexually transmitted infections (STIs) were significantly associated with oncogenic HPV infection, whereas higher smoking index and corticosteroid use were marginally associated with oncogenic HPV infection. In multivariate analysis, younger age (OR, 0.98 [0.96–0.99]), MSM (OR, 5.85 [2.33–14.71]), CD4 <100 (OR, 2.24 [1.00–5.01]), and having ≥2 STIs (OR, 2.81 [1.72–4.61]) were independently associated with oncogenic HPV infection. These 4 variables were also significant risk factors for multiple oncogenic HPV infection.

**Conclusions:**

Among Japanese HIV-infected patients, approximately two-thirds of MSM, one-fifth of heterosexual men, and one-fifth of women have anal oncogenic HPV infection. Younger *age*, MSM, ≥2 STIs, and immunosuppression confer a *higher risk* of infection with oncogenic HPV and multiple oncogenic types.

## Introduction

Human papillomavirus (HPV) infections are the most common sexually transmitted infections (STIs) worldwide[1]. HPV types that infect the anorectal area can be divided into oncogenic types and non-oncogenic types, and approximately 85% of anal cancers worldwide are attributed to oncogenic HPV[2,3]. In addition, infection with multiple HPV types has been associated with HPV persistence and longer duration of infection[4,5]; thus, co-infection with multiple oncogenic HPVs may be an important risk factor for anal cancer. Although anal cancers are rare in the general population[6], HIV-positive men who has sex with men (MSM) have an approximately 1.5 times higher prevalence of anal HPV infection and a 5 times higher risk of anal cancer than HIV-negative MSM[7,7–9], even in this era of highly active antiretroviral therapy (HAART) use[10].

Most data on anal HPV infection have been obtained in Western countries. A limited number of studies have reported the prevalence of anal HPV infection in Asia including Taiwan[7,11], India[12], Thailand[7], and China[7,8,13]. In Japan, 14,706 HIV and 6719 AIDS cases were reported at the end of 2012[14]. Although the prevalence of HIV in the general population remains low at 0.018%, the number of newly reported cases of HIV in MSM more than doubled from 314 in 2001 to 724 in 2012, and it is possible that the number of HIV-positive MSM could reach 10.4% in 2040[14,15]. Therefore, there have been concerns about the increasing trend of anal HPV infection as well as anorectal STIs such as syphilis[16] and invasive amebiasis[17]. However, no data are available on the prevalence of anal HPV infection in Japan. Prior data suggested an increase of anal HPV infection-associated anal intraepithelial neoplasia (AIN) in Asian MSM[18]. However, as it stands, most HIV-infected patients do not undergo anal screening in Japanese hospitals; thus, obtaining information on HPV prevalence and genotypes in Japanese HIV-positive patients is necessary for HPV-related AIN prevention.

Compared with HIV-positive MSM, few studies have reported the prevalence of anal HPV infections with different genotypes in HIV-positive heterosexual men or HIV-positive women[11,12]. In addition, only limited data are available on the risk factors for HPV infection in HIV-positive patients[9,19]. It remains poorly understood whether low CD4 counts, high viral load (VL), long duration of HIV disease, HAART use, or concomitant infection with STIs affect the risk of oncogenic HPV infection.

To address these issues, we prospectively collected information on oncogenic HPV infection as well as STIs and HIV-related factors, and we then determined the prevalence, genotypes, and risk factors for anal HPV infection in Japanese HIV-positive MSM, heterosexual men, and women.

## Material and Methods

### Study Design, Setting, and Participants

This prospective, cross-sectional study was carried out between September 2009 and March 2015 in the Department of Gastroenterology and Hepatology at the National Center for Global Health and Medicine (NCGM), Tokyo, Japan. Eligible patients were HIV-positive patients, at least 18 years old, who were willing to receive gastrointestinal (GI) tract cancer screening, provide anal swabs and blood for tests, and physically able and willing to provide written informed consent. NCGM has one of the largest HIV clinics in Japan with >3,500 registered patients as of May 2013. These patients were the first patients enrolled in the HIV-GI cohort (V-GI protocol) and were systematically screened for anal HPV infection and STIs during the study period. Written informed consent was obtained from all participants. This study was approved by the ethics committee of the National Center for Global Health and Medicine (Nos.1440) and was implemented in accordance with the provisions of the Declaration of Helsinki.

### Data collection

Blood samples were collected for syphilis, hepatitis B virus (HBV), *Chlamydia trachomatis* (*C*. *trachomatis*), and *Entamoeba histolytica (E*. *histolytica)*. Positive infection was defined as current or prior infection. Syphilis infection was defined as positive *Treponema pallidum* latex agglutination (TPHA) test and rapid plasma reagin (RPR) titer ≥8[20]. HBV infection was defined as positive hepatitis B surface antigen (HbsAg) or positive anti-HBs antibody; if HbsAg and anti-HBs antibody were negative, the patient was considered to be negative for HBV infection. In Japan, because universal vaccination against HBV has not been introduced and intervention to prevent mother-to-child transmission has been very successful[21], most adult cases with chronic HBV infection are considered to be sexually transmitted[22]. *C*. *trachomatis* infection was determined by anti-*C*. *trachomatis* IgG and IgA antibodies using an Enzyme-Linked ImmunoSorbent Assay (*C*. *trachomatis* IgA/IgG antibody, LSIM; LSI Medience, Tokyo, Japan). *C*. *trachomatis* infection was defined as positive IgA or IgG results; negative infection was defined as both negative IgA and IgG results. Amebic infection was assessed by anti-*E*. *histolytica* antibody (Ameba-Spot IF; bioMe´rieux, Marcy l’Etoile, France), as described previously[23]. Serum antibody titers <100 were considered negative, while titers of 100, 200, 400, 800, 1600, and 3200 were considered positive. The structured interview/questionnaire was completed on the day of HPV tests[24]. Patients were asked about i) their lifestyle habits (smoking history and alcohol consumption), ii) systemic steroid use for >2 weeks, iii) HIV-related factors including CD4 cell count, HIV-1 VL, duration (years) of HAART, and route of transmission (MSM/heterosexual infection, injection drug use, transfusion for hemophilia, and unknown) through face-to-face interviews by well-trained researchers in a private room[24]. Because the maximum period of supply for prescriptions is limited to 3 months in the Japanese health care system, patients need to make visits at least every 3 months for prescriptions as well as monitoring of CD4 cell count and HIV-1 VL.

### HPV detection and genotyping

A dedicated brush (DNAPAP cervical sampler, Qiagen, Gaithersburg, MD) was used for sampling of the anorectal area. The anal brush was inserted 3–5 cm into the anal verge and the dentate line and it was then used to scrape the anal walls by repeatedly rotating it clockwise and counterclockwise. Nucleic acids were extracted from 500-μL analsamples in the Sure Path solution with a commercial kit (QIAGEN DNA mini kit; Qiagen, Hilden, Germany)[25]. HPV DNA was genotyped by polymerase chain reaction (PCR)-Invader assay, as described previously[25]. This method could detect 14 oncogenic HPV genotypes (types 16, 18, 31, 33, 35, 39, 45, 51, 52, 56, 58, 59, 67, and 68)[25]. Study participants who were positive for any HPV type were considered to have a current HPV infection. A “multiple type” variable was created such that results at each sampling site were categorized as having 0, 1, or ≥2 oncogenic types. A participant was considered to have infection with multiple oncogenic types if ≥2 oncogenic types were detected at any of the sampled sites.

### Statistical analysis

Baseline characteristics were compared between patients with and without anal oncogenic HPV infection using the Mann–Whitney U test or χ^2^ test (or Fisher’s exact test) for continuous or categorical variables, respectively. We used logistic regression analysis to compute the odds ratios (ORs) and 95% confidence intervals (CIs) as an estimate of anal oncogenic HPV infection associated with clinical factors. For multivariate analysis, we used a multiple logistic regression model that included all factors with *p* <0.05 on univariate analysis (those included age <40 years, sex, MSM, CD4 <100, HIV VL >50,000, administration of HAART, and more than 2 STIs).

We also elucidated the association between infection with multiple oncogenic HPV types and clinical factors in uni- and multivariate logistic regression analyses. Sex was not included in the logistic regression model because all patients infected with multiple oncogenic HPV types were males.

The Cochran–Armitage test was used to determine trends in the proportion of oncogenic HPV infection according to CD4 count (<200, 200–399, 400–599, and ≥600), HIV VL (undetectable, 50–50,000, and >50,000), and duration of HAART (no administration, <5 years, 5–9 years, and ≥10 years).

Statistical significance was defined as two-sided *p* values <0.05. All statistical analyses were performed using Stata version 13 software (StataCorp LP, College Station, TX).

## Results

### Patient characteristics

During the study period, 421 HIV-infected patients were recruited. Patient characteristics are shown in Table 1. There were 395 (93.8%) men and 26 (6.2%) women, and their median age was 44 years. The majority of HIV infection was through anal intercourse (85.8%). Among HIV-positive patients, 18.3% had CD4 <100 cells/μL, 62.5% had an undetectable HIV VL, and 75.1% were receiving HAART with a median duration of 8 years. The rates of syphilis, HBV, *C*. *trachomatis*, and *E*. *histolytica* infection were 41.3%, 54.4%, 50.1%, and 26.1%, respectively. The proportion of subjects with ≥2 STIs was 58.2%.

**Table 1:** Characteristics of 421 HIV-infected patients.

Variables	n (%)	Median (IQR)
Age (years)		44 (39, 55)
Male sex	395 (93.8)	
Alcohol consumption		
None	208 (49.4)	
Light (1–50 g/week)	100 (23.8)	
Moderate (>360 g/week)	113 (26.8)	
Smoking index^†^		
Never smoker	164 (39.0)	
1–300	127 (30.2)	
>300	130 (30.9)	
**Comorbidities and drug use**		
Hypertension	54 (12.8)	
Diabetes mellitus	27 (6.4)	
Dyslipidemia	28 (6.7)	
Chronic kidney disease	9 (2.1)	
Chronic liver disease	94 (22.4)	
Corticosteroid use	25 (5.9)	
**HIV-related factors**		
Route of HIV infection		
MSM	361 (85.8%)	
Heterosexual	39 (9.3)	
Injection drug use	2 (0.5)	
Hemophilia	17 (4.0)	
Unknown	2 (0.5)	
CD4 cell counts (cells/μL)		369 (179, 582)
CD4 <100 (cells/μL)	77 (18.3)	
HIV VL (copies/mL)		51,500 (770, 320,000)
VL ≤50 (normal range)	263 (62.5)	
50< VL ≤50,000	79 (18.8)	
VL >50,000	79 (18.8)	
Administration of HAART	316 (75.1)	
Duration of HAART (years)*		8.0 (5.1, 11.8)
Duration ≤5 year	78 (24.7)	
5 years< duration ≤10 years	123 (38.9)	
Duration >10 yrs	115 (36.4)	
**Sexual transmitted infections**		
Syphilis infection	174 (41.3)	
Hepatitis B virus infection	229 (54.4)	
*Chlamydia trachomatis* infection	211 (50.1)	
*Entamoeba histolytica* infection	107 (26.1)	
Number of infections		2 (1, 3)
Number of infections ≥2	245 (58.2)	

Abbreviations: HAART, highly active anti-retroviral therapy; IQR, interquartile range; MSM, men who have sex with men; VL, viral load

*Duration of HAART was analyzed in 316 patients who had undergone HAART.

^†^The smoking index was evaluated in occasional and daily smokers and defined as the number of cigarettes per day multiplied by the number of smoking years.

### Prevalence of oncogenic HPV infection

The prevalence of anal HPV infection and the distributions of genotypes are shown in Table 2. The oncogenic HPV infection rate among the HIV-infected patients was 69.4%; specifically, it was 75.9% in MSM, 20.6% in heterosexual men, and 19.2% in women. The oncogenic HPV genotypes that were detected most frequently were as follows: HPV-58 (30.2%), HPV-16 (28.8%), HPV-52 (22.2%), and HPV-33 (18.8%) in MSM; HPV-16 (14.7%), HPV-31 (5.9%), HPV-33 (5.9%), and HPV-52 (5.9%) in heterosexual men; HPV-18 (7.7%), HPV-16 (3.9%), HPV-31 (3.9%), and HPV-33 (3.9%) in women. HPV-16/18 types were detected in 34.9% of MSM, 17.7% of heterosexual men, and 11.5% of women. The mean numbers of oncogenic HPV infection were 2.4 in MSM, 0.4 in heterosexual men, and 0.2 in women. Infection with multiple oncogenic HPV types was detected in 54.6% of MSM and 8.8% of heterosexual men, whereas HIV-infected women had no infection with multiple oncogenic HPV types.

**Table 2:** Anal oncogenic HPV infection prevalence and genotyping in HIV-infected patients.

	All HIV-infected patients (n = 421)	Men (n = 395)		Women (n = 26)
		MSM (n = 361)	Heterosexual (n = 34)	
Any oncogenic type HPV	286 (67.9)	274 (75.9)	7 (20.6)	5 (19.2)
16	110 (26.1)	104 (28.8)	5 (14.7)	1 (3.9)
18	42 (10.0)	39 (10.8)	1 (2.9)	2 (7.7)
31	58 (13.8)	55 (15.2)	2 (5.9)	1 (3.9)
33	71 (16.9)	68 (18.8)	2 (5.9)	1 (3.9)
35	53 (12.6)	53 (14.7)	0	0
39	45 (10.7)	45 (12.5)	0	0
45	32 (7.6)	32 (8.9)	0	0
51	57 (13.5)	57 (15.8)	0	0
52	82 (19.5)	80 (22.2)	2 (5.9)	0
56	38 (9.0)	38 (10.5)	0	0
58	110 (26.1)	109 (30.2)	1 (2.9)	0
59	76 (18.1)	76 (21.1)	0	0
67	56 (13.3)	55 (15.2)	1 (2.9)	0
68	38 (9.0)	38 (10.5)	0	0
16 or 18	135 (32.1)	126 (34.9)	6 (17.7)	3 (11.5)
Number of oncogenic HPV types, mean±SD	3.0±2.0	2.4±2.2	0.4±1.0	0.2±0.4
Multiple oncogenic HPV types	200 (47.5)	197 (54.6)	3 (8.8)	0

Abbreviations: MSM, men who have sex with men; SD, standard deviation.

### Risk factors for oncogenic HPV infection

Risk factors for oncogenic HPV infection are shown in Table 3. In univariate analysis, younger age, male sex, MSM, CD4 <100, HIV VL >50,000, no administration of HAART, and having ≥2 STIs were significantly associated with oncogenic HPV infection, whereas higher smoking index and corticosteroid use were marginally associated with oncogenic HPV infection. In multivariate analysis, younger age, MSM, CD4 <100, and having ≥ 2STIs were independently associated with oncogenic HPV infection.

**Table 3:** Risk factors for oncogenic HPV infection of the anorectal area (n = 421).

Variables	HPV (n = 286)/ without HPV (n = 135)	Crude OR (95% CI)	P value	Adjusted OR (95% CI)	P value
Age (years)	45.1±11.3/ 49.9±12.1	0.97 (0.95–0.98)	<0.001	0.98 (0.96–0.99)	0.024
Male sex	281 (98.3)/ 114 (84.4)	10.35 (3.81–28.12)	<0.001	1.23 (0.33–4.66)	0.759
Alcohol consumption, none	145 (50.7)/ 63 (46.7)	1 (reference)			
Light (1–50 g/week)	68 (23.8)/ 32 (23.7)	0.92 (0.55–1.54)			
Moderate (>360 g/week)	73 (25.5)/ 40 (29.6)	0.79 (0.49–1.29)	0.645		
Smoking index^†^, never smoker	105 (36.7)/ 59 (43.7)	1 (reference)			
1–300	96 (33.6)/ 31 (23.0)	1.74 (1.03–2.91)			
>300	85 (29.7)/ 45 (33.3)	1.06 (0.66–1.72)	0.086		
Hypertension	32 (11.2)/ 22 (16.3)	0.65 (0.36–1.16)	0.146		
Diabetes mellitus	15 (5.2)/ 12 (8.9)	0.57 (0.26–1.25)	0.159		
Dyslipidemia	17 (5.9)/ 11 (8.2)	0.71 (0.32–1.57)	0.399		
Chronic kidney disease	6 (2.1)/ 3 (2.2)	0.94 (0.23–3.83)	0.934		
Chronic liver disease	63 (22.0)/ 31 (23.1)	0.94 (0.58–1.53)	0.800		
Corticosteroid use	21 (7.3)/ 4 (3.0)	2.60 (0.87–7.72)	0.086		
Route of HIV infection, MSM	274 (95.8)/ 87 (64.4)	12.60 (6.40–24.79)	<0.001	5.85 (2.33–14.71)	<0.001
CD4 <100 (cells/μL)	66 (23.1)/ 11 (8.2)	3.38 (1.72–6.64)	<0.001	2.24 (1.00–5.01)	0.049
HIV VL >50,000 (copies/mL)	64 (22.4)/ 15 (11.1)	2.31 (1.26–4.22)	0.007	1.21 (0.50–2.96)	0.675
Administration of HAART	205 (71.7)/ 111 (82.2)	0.55 (0.33–0.91)	0.021	1.21 (0.59–2.49)	0.609
Number of STIs ≥2	200 (69.9)/ 45 (33.3)	4.65 (3.00–7.21)	<0.001	2.81 (1.72–4.61)	<0.001

Abbreviations: HAART, highly active anti-retroviral therapy; MSM, men who have sex with men; STIs, sexual transmitted infections; VL, viral load

^†^The smoking index was evaluated in occasional and daily smokers and defined as the number of cigarettes per day multiplied by the number of smoking years.

Risk factors for infection with multiple oncogenic HPV types are shown in Table 4. In univariate analysis, younger age, corticosteroid use, MSM, CD4 <100, HIV VL >50,000, no administration of HAART, and having ≥2 STIs were significantly associated with multiple oncogenic HPV infection. In multivariate analysis, younger age, MSM, CD4 <100, and having ≥2 STIs were independently associated with multiple oncogenic HPV infection.

**Table 4:** Risk factors for infection of the anorectal area with multiple oncogenic HPV (n = 335).

Variables	Multiple HPV (n = 200)/ without HPV (n = 135)	Crude OR (95% CI)	P value	Adjusted OR (95% CI)	P value
Age (years)	43.9±10.7/ 49.9±12.1	0.96 (0.94–0.97)	<0.001	0.97 (0.94–0.99)	0.008
Alcohol consumption, none	101 (50.5)/ 63 (46.7)	1 (reference)			
Light (1–50 g/week)	52 (26.0)/ 32 (23.7)	1.01 (0.59–1.74)			
Moderate (>360 g/week)	47 (23.5)/ 40 (29.6)	0.73 (0.43–1.24)	0.456		
Smoking index^†^, never smoker	73 (36.5)/ 59 (43.7)	1 (reference)			
1–300	68 (34.0)/ 31 (23.0)	1.77 (1.03–3.06)			
>300	59 (29.5)/ 45 (33.3)	1.06 (0.63–1.78)	0.095		
Hypertension	20 (10.0)/ 22 (16.3)	0.57 (0.30–1.09)	0.091		
Diabetes mellitus	10 (5.0)/ 12 (8.9)	0.54 (0.23–1.29)	0.164		
Dyslipidemia	13 (6.5)/ 11 (8.2)	0.78 (0.34–1.81)	0.567		
Chronic kidney disease	4 (2.0)/ 3 (2.2)	0.90 (0.20–4.08)	0.889		
Chronic liver disease	40 (20.0)/ 31 (23.1)	0.83 (0.49–1.41)	0.493		
Corticosteroid use	19 (9.5)/ 4 (3.0)	3.44 (1.14–10.34)	0.028	3.04 (0.81–11.5)	0.100
Route of HIV infection, MSM	197 (98.5)/ 87 (64.4)	36.23 (10.98–119.49)	<0.001	8.07 (2.23–29.3)	0.001
CD4 <100 (cells/μL)	53 (26.5)/ 11 (8.2)	4.06 (2.03–8.12)	<0.001	2.86 (1.21–6.76)	0.017
HIV VL >50,000 (copies/mL)	49 (24.5)/ 15 (11.1)	2.60 (1.39–4.86)	0.003	1.51 (0.56–4.05)	0.413
Administration of HAART	136 (68.0)/ 111 (82.2)	0.46 (0.27–0.78)	0.004	1.30 (0.58–2.93)	0.525
Number of STIs ≥2	144 (72.0)/ 45 (33.3)	5.14 (3.21–8.25)	<0.001	3.11 (1.78–5.43)	<0.001

Abbreviations: HAART, highly active anti-retroviral therapy; MSM, men who have sex with men; STIs, sexual transmitted infections; VL, viral load

^†^The smoking index was evaluated in occasional and daily smokers and defined as the number of cigarettes per day multiplied by the number of smoking years.

**Fig 1:**
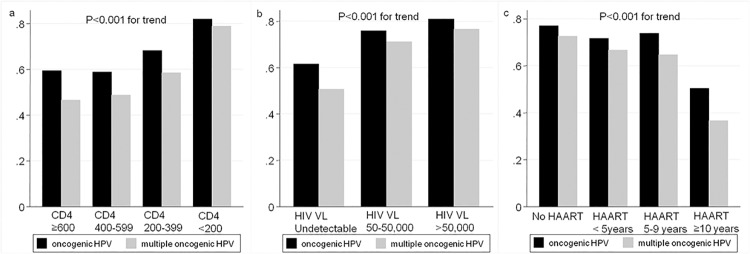
Anal HPV infection rates associated with HIV-related factors. Abbreviations: VL, viral load; HAART, highly active anti-retroviral therapy.

Oncogenic and multiple oncogenic HPV infection rates tended to increase with the decrease in CD4 (*p*<0.001 and *p*<0.001, respectively; Fig 1A) and with HIV VL increase (*p*<0.001 and *p*<0.001, respectively; Fig 1B), while they tended to decrease with longer HAART duration (*p*<0.001 and *p*<0.001, respectively; Fig 1C).

## Discussion

In regard to the prevalence of anal HPV infection, we found that 76% of HIV-infected MSM had an oncogenic HPV type, which was similar to the rates reported in other Asian countries. In Shenzhen, China, 71% of HIV-positive MSM had HPV infection[8], while in Beijing, 61% of HIV-positive MSM had oncogenic HPV infection[26]. In Taiwan, 40–64% of HIV-infected men had oncogenic HPV infection[7,11]. In Bangkok, Thailand, 58% of HIV-positive MSM had oncogenic HPV infection[7]. The anal HPV prevalence in our study was lower than the reported 81–92% in Western countries among HIV-positive men[19,27,28].

Few studies have investigated anal HPV infection in HIV-positive heterosexual men and women. In our study, the prevalence of anal oncogenic HPV infection among HIV-positive heterosexual men was 21%. Christophe et al investigated 50 HIV-positive heterosexual male injection drug users with no history of anal intercourse and 44% had oncogenic HPV[29]. In the study of 64 HIV-positive heterosexual men by Chien et al., 38% had anal oncogenic HPV infection[11]. The mechanisms of anal HPV infection in the absence of anal intercourse are not well known, but infection is possibly due to insertion of transiently infected fingers or toys, as well as shedding from other infected genital sites[29], and thus, anal HPV infection may behave as a STI. The reason for the low prevalence of infection among HIV-positive heterosexual men in our study compared with the previous two studies is that 71% of the heterosexual men were hemophilia patients who were infected with HIV through treatment with blood products or blood transfusions[30] and not sexually. In our study, 19% of HIV-positive women had oncogenic HPV infection. In India, 9% of HIV-positive women had anal oncogenic HPV and 17% had oncogenic cervical HPV infection[12]. These findings, and the fact that cervical cancer increases the risk of anorectal cancer in women[31], suggest the potential utility of both cervical and anal cancer screening of HIV-infected women for HPV infection of either the cervical or anal area, or both.

A wide range of HPV types was detected in the anal canal of HIV-positive patients. In this study, the single most frequently detected type was HPV-58 (30.2%) in HIV-infected MSM. Supindham et al. recently reported that HPV-58 infection rate was 29% in HIV-infected MSM in Northern Thailand [32], which is quite similar to the rate in our study. They also showed a lower rate of HPV-58 (18%) in non-HIV-infected MSM compared with HIV-infected MSM [32]. In another study in Bangkok, Phanuphak et al reported that the HPV-58 infection rate was significantly higher in HIV-positive MSM (10.8%) than in HIV-negative MSM (4.9%) [33]. These results suggest that there might be a positive association between HIV and HPV-58. Fife et al. reported that HPV types 51, 52, 56, and 58 are among the types that lead to cervical dysplasia or cancer in the presence of HPV-16 [34]. Thus, it is possible that co-infections with HPV58 and HPV16 or 18 are more likely to cause AIN compared with infection by a single HPV type. On the other hand, HPV types 16 and 18 in MSM were detected in 29% and 11%, respectively, which are in agreement with other studies of Asian HIV-positive MSM. Specifically, the rates for types 16 and 18 in previous studies were 16% and 11% in Beijing, China; 34% and 14% in Beijing and Tianjin, China; and 10% and 8% in Taiwan, respectively. For a given HPV type in the bivalent HPV vaccine, 35% of MSM were infected with the same HPV type in our study. Because the HPV vaccine appears to be safe in HIV-positive men[35], a significant proportion of HIV-positive MSM in Japan may potentially benefit from the vaccine, which might prevent HPV-associated AIN.

Co-infection with multiple oncogenic HPV types has been associated with persistent infection and infection with a longer duration, and it thus represents an important risk factor for anal cancer[2,4,5]. We found that infection with multiple oncogenic HPV types was detected in 54.6% of MSM. The prevalence rates of infection with multiple HPV types in HIV-positive men have been reported to range widely, namely, between 6% and 61%. The difference in rates was thought to be due to the different number of HPV types that are detected by different HPV sample kits. In agreement with two studies that showed 5–10% of HIV-positive heterosexual men were infected with multiple HPV types in the anal canal[11,29], our study revealed that 9% had multiple oncogenic HPV infection.

Previous studies have shown specific risk factors for anal HPV infection including younger age[36,37], HBV infection[38], and positive *C*. *trachomatis* serology[39]. These findings suggested that sexual transmission has an important role in anal HPV infection. Consistent with these findings, multivariate analysis in our study showed that younger age, MSM, and having ≥2 STIs were independently associated with infection by any oncogenic HPV type and multiple oncogenic HPV types. In addition, we found that anal infection with oncogenic and multiple oncogenic HPV types was significantly associated with decreased CD4 counts, high HIV RNA, and short HAART duration, and CD4 <100 was an independent risk factor. Some[9,11,37–40], but not all[9,11,38], studies found increased anal HPV infection among HIV-positive men with decreased CD4 counts. Nishijima et al showed that HPV types 16 or 18, low CD4 cell count, and current smoking are associated with an increased risk of anorectal condyloma in HIV-infected patients [41]. These findings and our data indicate that immunosuppression is an important risk factor for anal HPV infection.

There are several limitations to this study. Although we collected information on syphilis, HBV, *C*. *trachomatis*, and *E*. *histolytica* infection, information on the number of sexual partners, receptive anal intercourse, condom use, and *Neisseria gonorrheae* infection was not collected. In addition, the number of subjects in this study was relatively large (n = 421), but the study population was a convenience sample of patients attending the Department of Gastroenterology; therefore, it remains unknown if this sample population is representative of the general HIV-positive community.

In conclusion, this prospective, cross-sectional study of Japanese HIV-infected patients demonstrated that approximately two-thirds of MSM and one-fifth of heterosexual men and women had anal oncogenic HPV infection. In the risk analysis, younger age, MSM, CD4 <100, and ≥2 STIs were independently associated with oncogenic HPV infection and multiple oncogenic HPV types. Our findings support the need for establishing an anal cancer screening system and regular follow-up strategies, especially in HPV high-risk HIV-positive patients, for preventing HPV transmission and detection of early anal neoplasia in Asia as well as Western countries.
